# Syntactic change in diachrony versus contact-induced change: two sides of the same coin?

**DOI:** 10.1515/tlr-2025-0012

**Published:** 2025-11-18

**Authors:** Roberta D’Alessandro, Michael T. Putnam, Silvia Terenghi

**Affiliations:** Utrecht University, Utrecht, Netherlands; Penn State University, Pennsylvania, USA; University of Greenwich, London, UK

**Keywords:** language contact, *ϕ*-features, discourse features, indexicals, clitics, differential object marking (DOM)

## Abstract

Recent studies (e.g. Kupisch, Tanja & Maria Polinsky. 2022. Language history on fast forward: Innovations in heritage languages and diachronic change. *Bilingualism: Language and Cognition* 25. 1–12) have rekindled an old debate concerning whether language change in contact (CIC) and language change in diachrony (CID) proceed along the same developmental path, or whether they diverge from one another in fundamental and predictable ways. This paper contributes to this ongoing debate; we propose a new heuristic to determine similarities and differences in syntactic change in CIC and CID. We postulate that the primary distinction boils down to the type of features related to the domain of syntax under investigation, i.e., situations involving (formal) *ϕ*-features lead to similar trajectories of change in both CIC and CID, while those driven by discourse-features show divergence. We test our hypothesis on a host of different empirical data, e.g., indexicals, (subject) clitics, and differential object marking (DOM) as evidence for our claim.

## 1 Introduction

A question that has often come to the forefront in research on historical and contact linguistics circles, and that has recently garnered much attention once again, concerns the similarities and differences between language change in diachrony (CID) and language change in contact (CIC). To put it bluntly, exactly how distinct or how similar are the outcomes of syntactic change across these areas of linguistic research when directly compared with each other? Although some linguists explicitly claim that CIC and CID, i.e., diachronic, endogenous change, basically proceed along the same lines (cf. Heine and Kuteva 2005), others highlight their differences (cf. among others Thomason and Kaufman 1988). In general, this issue has been addressed in many venues, especially in typological studies (Haase and Nau 1996) and creole studies (e.g., Bickerton 1981; Michaelis and Haspelmath 2020). Recently, Kupisch and Polinsky (2022) have included heritage languages, i.e., L1s acquired in a restricted context, one where the larger community uses a different language, in the pool of contact situations to be considered for this comparison, claiming that “grammatical patterns in heritage languages can be predicted on the basis of diachronic change” (Kupisch and Polinsky 2022: 2).

This paper intends to contribute to this debate by exploring to what extent CID on the one hand and CIC on the other are similar or distinct from each other within the realm of morphosyntax. Concretely, we propose a new heuristic responsible for establishing a typology of *similarities* and *differences* that exist between these two types of change. We do so by drawing a distinction between core grammar phenomena (which exclusively rely on sem(antic)-syn(tactic) features) and interface phenomena (which instead rely on the requirements of different modules of grammar). Although this is not innovative *per se* (see Sorace 2011 for the first fully-fledged development of this logic applied to L2 acquisition), our contribution lies in its application to the domain of language change. As we argue in this work, the distinction between core grammar phenomena and interface phenomena grants us a more fine-grained window into the question of whether CID and CIC might be reduced to the same underlying mechanisms and allows us to make specific predictions as to when the two will show some degree of overlap as opposed to when they will not show any isomorphism.

Against this background, we maintain that the claim that outcomes of CIC and CID are overwhelmingly the same, so much so that the former can straightforwardly be predicted on the basis of the latter, and that CIC “can amplify and foreground developments that are known to take place in language diachrony” (Kupisch and Polinsky 2022: 2), is only true when functional features are the target of change, and much less obvious when the elements undergoing change are discourse-oriented. We illustrate this dichotomy by examining a number of case studies and highlighting the output of change for one and the same construction both in isolation (CID) and in contact (CIC), following mostly the *microcontact* methodology (D’Alessandro 2015, 2021). We show that while change affecting formal (*ϕ*-)features is rather predictable and follows a well-defined path, which is the same in contact and in diachrony, CIC can have radically different outputs from CID when discourse or pragmatic elements are involved. For the sake of time and space, we restrict our discussion of ‘formal’ features in this manuscript to *ϕ*-features, with a secondary focus on case and discourse features, such as topic and focus.1We thank an anonymous TLR-reviewer from bringing this to our attention. Future, more expansive and detailed work on differences and similarities between CID- and CIC-processes requires the treatment of a wider array of formal features.

This paper is structured as follows. In the following section (Section 2), we provide the theoretical background against which we will carry out our comparison between syntactic CID and CIC. In Section 3, we sketch out the key similarities and differences held to exist between syntactic change in CID- and CIC-environments and flesh out our proposal in greater detail. Section 4 is devoted to the investigation of pronouns and demonstratives, for which we show that CID and CIC indeed converge. Section 5 introduces data for DOM, which show that the outcomes of syntactic change in contact only partially overlap with those of CID, indicating that the two are not of the same nature. Finally, Section 6 illustrates an empirical domain, that of subject clitics, where CIC is most radically different from CID.

## 2 Theoretical preliminaries

Before addressing the direct comparison between CIC and CID, it is necessary to establish both the heuristic of comparison and exactly which elements of syntax proper are being compared with one another. One of the primary goals of this paper is to show that talking about syntactic change, be it in diachrony or in contact, without defining the target of this change is fraught with trouble because the various generalizations will not be drawn with sufficient precision. To start, we assume a modular model of grammar, with syntax being one of the modules (or sub-components) of grammar. Syntax is the locus where functional features interact, to produce a complex expression which requires interpretation at the interfaces involved in decoding these expressions for sound and meaning (i.e., LF and PF). Furthermore, we assume a post-syntactic lexical insertion model, according to which the terminal nodes in syntactic structure are not morphemic (or fully-fledged lexical items) (Baunaz et al. 2018; Borer 2013; Caha et al. 2025; Embick 2015; Embick and Noyer 2007; Halle and Marantz 1993; Starke 2009), but features or feature bundles. We will not comment further on the differences found among different lexical insertion models. One facet they all share is the call for a disassociation of formal features from exponents, which is a position we make use of in the pages that follow.

Here, we draw a distinction between (i) operations targeting *ϕ*-features and (ii) operations involving other modules, such as pragmatics. As previously established in the literature, most notably Sorace (2011) and Hulk and Müller (2000) among many others, structures involving interfaces are more difficult to process; they are also more prone to errors in L2 acquisition, precisely because the speaker needs to meet well-formedness requirements in their grammar for multiple modules. For example, for a speaker to decide whether a sentence can be null-subject or not, many different pieces of information will have to come together: syntactically, the language will have to allow *pro*-drop; morphologically, the verbal agreement associated with this syntactic operation will most likely be tied to richly inflected morphological paradigms, and the language will have to license and realize, or pronounce, a null pronoun with certain characteristics. Syntax and morphology are not the only modules involved in the computation and expression of null subjects: the speaker needs to meet the appropriate discourse conditions, ensuring that the information about the subject’s referent can be retrieved from a silent element. In short, this process of producing and/or comprehending a ‘silent’ syntactic objects requires the interaction of many different modules, which in turn determines the larger number of deviant sentences in L2 speakers, as well as heritage speakers.

We maintain here that this distinction between syntax-internal operations targeting *ϕ*-features and interface-targeting operations must be considered when investigating specific instances of language change, too. Specifically, we argue here that change affecting features within the narrow syntax is more easily predictable and constrained. In this respect, research focusing on elements of diachronic and contact-driven change gives the impression that CID and CIC are one and the same process, or at the very least, that they are closely related. We hypothesize that this is, in fact, the case; they have the same output and predictability, when *ϕ*-features are subject to change. In contrast, when more than one module is involved in the change, the outcomes of this change will be less predictable.

## 3 Syntactic change in diachrony and in contact

To ground our nuanced heuristic of comparison more robustly, in this section we provide a more detailed discussion of the literature on syntactic change in diachrony and in contact (Section 3.1). We then proceed to spell out the methodological challenge that underlies any attempt to compare CID and CIC and introduce the Microcontact approach as a solution to the issue (Section 3.2). This allows us to further elaborate on our heuristic for predicting similarities and differences across the outcomes of CID and CIC (Section 3.3).

### 3.1 A brief comparison

Syntactic change, and language change more broadly, might well constitute one of the most baffling facts for our linguistic theories. Syntactic acquisition simply amounts to the “transmission of syntactic systems from one generation to the next” (Willis 2017: 491), which implies that any given system must have been acquirable, and in fact fully acquired, by previous generations. We therefore need to explain why and how the transmission of a given system substantially breaks down at one of its given iterations. A variety of factors have been proposed over time to account for the observed changes: these may be reduced to factors endogenous to the (part of the) system that undergoes change (spontaneous innovation) and to a heterogeneous host of exogenous factors, which can all be ultimately traced back to some fundamental mutations affecting the acquisition process and its original conditions. These include, among others, specific input-related alterations (e.g. whether the input is timely or delayed; and whether it is sufficient or restricted), as well as the possibility that the grammatical system undergoing change exists side by side with one (or more) additional systems.2For an overview of the relevant factors, the reader is directed to Willis 2017; Bowern 2008. While the latter class of factors is clearly relevant to research in the field of contact linguistics and, more recently, of heritage linguistics, diachronic strands of research have honed in on the investigation of endogenous factors.

This considerable shift in focus becomes apparent as soon as we seek to compare the main insights into CID and CIC as presented in the relevant literature. Universals of language change (Haspelmath 1999; Hopper and Traugott 1993; Lehmann 1993; Meillet 1912) and the directionality of change (Kiparsky 1968; Hopper 1991; Keller 1994; and more recently Newmeyer 1998; Campbell 2001; Traugott 2001; Haspelmath 1999, 2004) are central to CID-studies. Generally speaking, the key process on which many descriptions of CID concentrate is simplification (or loss) (see Haspelmath 1999 for an overview), which is very often expressed in terms of *grammaticalization*. Grammaticalization, like its opposite, *degrammaticalization* (Norde 2009), takes place along a path; the identification of this path makes diachronic change relatively predictable. Theoretically oriented work on CID also focuses mostly on the grammar-internal causes and mechanisms of language change (Roberts and Roussou 2003; Roberts 2007; Roeper 1993; Lightfoot 1991; Kroch 1994; Lightfoot and Westergaard 2007; Westergaard 2008, 2011; Lohndal and Putnam 2024, a.o.).

In contrast, typological studies (see Koptjevskaja-Tamm 2010 for an overview), especially those focusing on language change in multilingual contexts (second language acquisition, heritage linguistics, creole linguistics), have highlighted the fact that CIC seems to be much more unpredictable than CID, since the former, unlike the latter, does not seem to follow a similar specific path. This is commonly assumed to stem from the observation that CIC results from the interplay between grammatical and facilitating factors that are external to the grammar. For instance, Aikhenvald (2006) provides a detailed list of factors that have an impact on lexical or grammatical borrowing in contact situations. Among the grammatical factors are pragmatic salience, tendency to achieve word-for-word inter-translatability, the existence of a perceivable gap in a paradigm, typological naturalness, pre-existing structural similarity, the existence of a lookalike; among extra-grammatical factors, she lists the degree of knowledge of each other’s languages (*lingualism*), the kind of contact languages are in, the attitudes of the speakers toward the languages, whether contact is balanced (i.e. the languages have an equal social status) or displacive, complete or incomplete language acquisition, and many others.

This complex picture for CIC is considered by some to significantly hinder our collective predictive capabilities, to the extent that, while simplification is commonly seen as the hallmark of diachronic change, both simplification *and* complexification are attested in contact settings.3As an anonymous TLR-reviewer correctly observes, equating simplification to grammaticalization in the context of diachronic change is itself an oversimplification. Reasons of space prevent us from doing full justice to the debate here; we therefore refer the interested reader to Hopper and Traugott (1993); Roberts and Roussou (2003); Dahl (2011). Any restrictive, formal model of grammar is capable of accounting for grammatical representations that ‘shrink’ and ‘expand’; however, what is desired, if attainable, is a systematic account pertaining to *when* and *why* these changes may occur. More acutely, the formal challenge is to deduce whether or not properties of composition of linguistic representations or underlying cognitive factors promote or hinder these instances of ‘shrinking’ and ‘expanding’ in both CIC- and CID-contexts (Aboh 2015; D’Alessandro and Frasson 2023; Pakendorf 2022).

### 3.2 A methodological challenge

At this juncture, it is worth mentioning that the very assumption that CID and CIC should be considered separate processes (as one could rightfully infer from the dichotomic literature on syntactic change) is not obvious. Languages, by default, are constantly undergoing some degree of change: this is true even when looking at the language development of a single speaker across their lifespan (see Sankoff 2018 for a general overview; see also D’Alessandro et al. 2025; Putnam and Natvig 2025 for reflections that also appeal to the notion of language contact across the lifespan). Furthermore, no language is an island, and in fact every language is usually in contact with many others; contact can well last for several centuries. The most remarkable case in point is provided by linguistic areas (*Sprachbünde*, Trubetzkoy 1930; for an overview, see van Gijn and Wahlström 2023), but this observation applies more generally; in fact, as noted by Bowern (2008), first language acquisition itself could rightfully be viewed as an instance of language contact, that between the child and the speakers that provide them with linguistic input. As a result, operationally disentangling CID from CIC is virtually impossible.

If we consider what we can call the extreme cases of CID and CIC, for instance by examining languages that have been in isolation for many centuries, like Icelandic on one side, and languages emerging from very intense and time-restricted contact, like creoles on the other, we can consider CIC as a separate phenomenon from CID. However, what we cannot control, even in these cases, are the socio-historical factors that might enhance or cause the change. Ideally, the same situation needs to be replicated in many possible contexts, through changing the dyads involved, and through the parallel observation of the language change in isolation. This is possible only in unique circumstances, i.e. when multiple instances of contact take place between the same language dyads at the same time and under the same socio-historical circumstances. One such instance is the case of Italo-Romance heritage languages in the Americas: after WW2, a massive emigration from Italy to North and South America took place. The emigrants were mostly monolingual speakers of one Italo-Romance variety (but not Italian), they left at the same time and relocated to a far-away land, severing their contacts with Italy. This has given a unique open-air laboratory to control for some features in contact with multiple Romance varieties (see Andriani et al. 2022a for a detailed description of the sociolinguistic conditions of the various communities).

**Figure 1:**
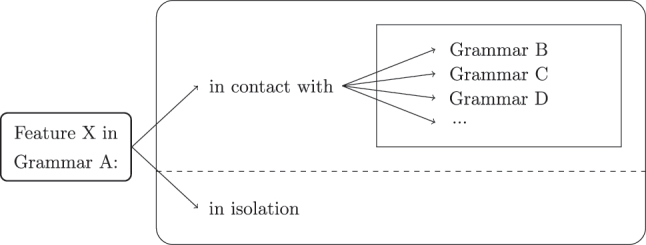
The microcontact methodology.

The *microcontact* methodology (D’Alessandro 2015, 2021), which consists in isolating one specific feature and checking its development in contact with multiple other languages of the same family, has been designed precisely with the aim to disentangle various possible co-occurring factors affecting the outcomes of change; in particular, we employ it to ascertain whether an attested instance of syntactic change should be attributed to contact or not. The basic idea is to consider nearly identical grammars (ideally, genetically related languages) differing in only one feature (call it X) in a given domain, and observe the development of that feature in a given grammar (call it A) in contact with all other grammars of the set, and in that grammar in isolation. If the feature X of grammar A shows divergent paths of change in contact with different grammars, as well as in isolation, we can attribute its change to contact (factoring out all the possible sociolinguistic elements that could have an impact on this change). If the development is identical in contact with all several different grammars, as well as in isolation, we attribute this change to an endogenous cause. This approach is shown schematically in Figure 1.

In what follows, we will primarily examine data elicited with the microcontact methodology as applied within the Romance family. Two notes are in order: firstly, as remarked above, languages are hardly ever isolated. Despite never having been completely isolated, Romance languages exhibited a good degree of isolation in the past; more importantly, they display some macroparameters (following Ledgeway’s 2012 classification) which are shared by all or most languages in the family. We take these common features to be an excellent source of information concerning endogenous change. As an example consider auxiliary selection. Latin did not have auxiliary selection in the way that Romance languages have it: it used be for all periphrastic forms of the verb (chiefly: passives); have was instead not used as an auxiliary. On the contrary, all present-day Romance languages have some form of have as auxiliary (with the exception of Portuguese, which has *ter* ‘to have’, but from the original Latin tenere ‘to hold’): given its presence across Romance, we regard this as an internal development.

Secondly, as it often happens with heritage language data, it was not always possible to find exact minimal pairs, due to the enormous variability in HL. Furthermore, despite all our efforts, we were not always successful when trying to minimize the differences between speakers and their social and linguistic background. The profile of some heritage languages is such that it does not allow to find large an uniform groups of speakers (see also Rothman and Treffers-Daller 2014; Leivada et al. 2019; D’Alessandro et al. 2021). Despite all the limitations of this domain of research, some solid generalizations can be drawn; we will discuss those, with the disclaimer that the data are at times fragmented or incomplete.

In this paper we investigate both the validity of this approach and the robustness of its coverage with respect to some morphosyntactic phenomena.

### 3.3 A new heuristic

Kupisch and Polinsky (2022) take a similar comparative stance to assess whether CIC and CID are underlyingly the same: concretely, they examine the grammaticalization path of demonstratives and numerals into articles both in heritage languages, one of the possible change in contact scenarios, and in diachrony. Based on their survey, they conclude that CIC and CID are essentially the same, as the syntactic patterns of change found in contact are the same as those that are found in diachronic change. Further, they argue that our knowledge of diachronic change (and, more specifically, of grammaticalization patterns) enables us to make predictions about syntactic change in contact, the only difference possibly being the pace of the relevant change, as CIC amplifies the effects of CID (to paraphrase them). More directly, syntactic change in contact can essentially be equated to accelerated diachronic change.

The thrust of our argument is that, although the position staked out by Kupisch and Polinsky (2022) holds for a number of cases, the overall picture is admittedly more complex. As a result, the proposal requires refinement. Concretely, we show that there are cases in which CIC *cannot* be straightforwardly reduced to accelerated diachronic change and, in turn, *cannot* be fully predicted on the basis of the attested outcomes of diachronic change. Instead, we show that different types of grammatical elements (that is to say: features) react differently to the pressure of change, and therefore they need to be considered separately when examining change. We substantiate this proposal by reviewing different syntactic phenomena: pronouns and demonstratives, Differential Object Marking (‘DOM’), and subject clitics. While the former two phenomena are purely structural, grammatical phenomena, the latter two can be classified as being ‘interface phenomena’, in connection with certain discourse-pragmatic requirements that they need to obey. We show here that Kupisch and Polinsky’s conclusions hold for the first domain, but not for the second one, where the outcomes of syntactic change in contact and of syntactic change in diachrony exhibit divergent paths of development (see also Sorace 2011 for similar considerations on the different nature of phenomena yielding different results in contact/L2 situations).

We relate this difference across domains to different third-factor strategies handling the relevant grammatical elements at play. Specifically, we propose that changes that affect *ϕ*-features hinge on a cognitive bias towards monotonic computations (Terenghi 2023b) which ensures that their change in contact converges with their change in diachrony, allowing for full predictability of such changes. In contrast, changes that affect discourse-related features, and in particular elements that are related to topicality, or linking with discourse, show important divergences across contact and diachrony. It is therefore possible to predict language change for purely syntactic features; however, this predictability is hindered in contact situations involving different kinds of features, such as semantic or discourse-related features. In case these types of features are involved, the output of CIC might be radically different from that of spontaneous CID, which we take to be monodirectional and predictable, when clarity of initial conditions exists. In a sense, this article is an integration of what Sorace (2011) and all subsequent work on the Interface Hypothesis, up to the recent Hoot and Leal (2023) have already pointed out about contact. Our contribution consists in checking the claims of “predictability” against what is a clearer source of predictable change, i.e. endogenous diachronic change.

## 4 Indexicals: CIC equals CID

In this section, we consider two classes of person indexicals: personal pronouns and (ternary) demonstrative systems. In particular, we focus on the semantic oppositions that are encoded in each of these systems, that is: how many person categories are available in a given language and whether these undergo any change (loss of erstwhile semantic contrasts, emergence of new semantic contrasts). Importantly, and despite the fact that indexical elements are clearly linked to the wider extra-linguistic context, from which they get their interpretation, we regard the oppositions relevant here as syntactically encoded by means of person features (following standard approaches to personal pronouns: Harley and Ritter 2002; Déchaine and Wiltschko 2002). As such, indexicals are considered here as purely grammatical elements; as a consequence, any change (or lack thereof) in this domain is regarded as fully syntactic matter, with no pragmatic or discourse-related ramifications.

### 4.1 Pronouns: no change

In diachrony, the organization of pronominal paradigms is mainly stable. This has been perhaps most famously stated by Nichols (1992), who concluded, on the basis of investigations carried out over a typologically diverse and balanced linguistic sample (*n* = 167), that the encoding of the inclusive/exclusive distinction (that is, both its presence and its absence) in pronominal paradigms is remarkably stable genetically: “pronouns and pronoun categories tend to be conservative in families” (Nichols 1992: 123–124). Stated differently, pronominal paradigms that originally displayed three distinct person categories (the speaker, the hearer, the other(s)) typically retain their ternary partition through the diachronic development of the given language; likewise, paradigms that originally displayed four different person categories (i.e., that make the inclusive-exclusive distinction in the first person) also typically maintain this quaternary partition through time.

As a concrete example, consider Table 1. As Table 1 shows, in spite of the apparent degree of morphological differentiation across the two varieties, Campidanese Sardinian fully preserves the ternary partition of the Latin pronominal system: that is, in both varieties, three persons are contrastively encoded.4Morphological differentiation within the paradigm is orthogonal to the issue under discussion (here, see in particular both Campidanese 3rd person forms, which are etymologically distinct from their Latin counterparts; and Campidanese 2pl form, which is a morphologically complex form: literally, ‘you others’). A comprehensive overview of such variation in the diachrony of Romance languages is provided by Cappellaro (2016).

**Table 1:** Pronominal paradigms in diachrony: from Latin to Campidanese Sardinian (Mensching and Remberger 2016: 278).

	1sg	2sg	3sg	1pl	2pl	3pl
Latin	ego	tu	ille	nos	vos	illi
Campidanese	deo	tu	issu	nosu	bosaturus	issos

Likewise, pronominal paradigms are generally held to be largely stable in contact settings (Heine and Kuteva 2005; Matras 2009; a.o.). In these contexts, the occasional (lexical or structural) borrowings may give rise to a new category or to the loss of an old one (that is, to change from ternary to quaternary systems or from quaternary to ternary systems, respectively), but such changes are considerably rare, as discussed by Siewierska (2004: chapter 7.3).

The stability of the indexical oppositions encoded in pronominal paradigms in contact settings can be shown by considering creole languages. Data relative to some of these varieties are collected in the *Atlas of Pidgin and Creole Language Structures* (*APiCS*; Michaelis et al. 2013). Relevant for the present discussion is APiCS’ feature 15, *Inclusive/exclusive distinction in independent personal pronouns* (Haspelmath et al. 2013), which surveys the structure of the pronominal paradigms of 75 creole languages. For our purposes, these should be compared with the pronominal paradigms of the respective lexifiers (i.e., the variety that provided the lexicon) and substrates. Out of 75 creoles, 64 straightforwardly retained the structure of the pronominal paradigms of their lexifiers (a ternary system in 61 cases, as shown in Table 2 for Portuguese-based Korlai; a quaternary one in the remaining 3 cases) and 10 more retained the distinctions made in their substrates (5 creoles display the inclusive/exclusive distinction, although their lexifier does not; 5 creoles do not display the inclusive/exclusive distinction, although their lexifier does). Only one creole (Sranan) introduced the inclusive/exclusive distinction from scratch, that is, without having it in its constitutive pool of features.

**Table 2:** Pronominal paradigms in contact: Portuguese-based Korlai (Clements 2013).

	1sg	2sg	3sg	1pl	2pl	3pl
Portuguese	eu	tu	ele	nós	vós	eles
Korlai	yo	ʋɔ	el	nɔ	udzo	elo

In summary, and again barring orthogonal lexical or morphological differences,5For instance, in the Korlai case in Table 2, 2sg is etymologically related to Portuguese 2pl (the original Portuguese form underwent a change in its number features, but crucially not in its person features). Moreover, Korlai 2pl and 3pl are not the simple reflexes of the original Portuguese forms; they are instead morphologically complex forms which combine the lexifier’s pronouns with the form *outro* ‘other’ (Bollée and Maurer 2016: 453). the structure of pronominal paradigms in contact contexts such as in creole languages is quite stable, with an overall preference for the preservation of the distinctions originally encoded in the lexifier and, to a lesser extent, the adaptation of the lexifier’s paradigm to accommodate the paradigmatic structure of a substrate variety. Only very rarely does the pronominal system change by drifting away from its pool of features.

The foregoing showed that, in general terms, the indexical oppositions encoded in pronominal paradigms tend to be stable both across time and, albeit slightly less so, in contact settings. In the latter case, however, departures from the original paradigms are commonly explained as an effect of substrate influence. With this disclaimer in mind, it can be safely concluded that there is little to no room for change in pronominal paradigms and, in this sense, diachrony and contact behave in a similar fashion.6An anonymous TLR-reviewer wonders why it is that pronominal paradigms are heavily resistant to change. While we do not have a fully-fledged explanation for this state of affairs (which is however well acknowledged in historical linguistics, where pronominal systems are commonly regarded as indicative of language familiarity), we would like to suggest that resistance to change might be linked to the limited room for variation that pronominal paradigms afford: grammatical person is mapped to an ontology that is rather limited in size (the speaker, the hearer, and the others; see e.g. Harbour 2016) and as such we might hypothesize that the very possibility of departures from the mappings available in the input is very constrained.

### 4.2 Demonstratives: CID and CIC have the same outputs

Let us now turn to a different class of indexical elements; namely, demonstrative systems. Although these are traditionally understood as spatial indexicals (Bühler 1934; see also Lyons 1977; Levinson 1983: chapter 2; Fillmore [1971] 1997; Diessel 2012; a.o.), in what follows we regard them as ultimately akin to person indexicals (such a personal pronouns) by virtue of their being derived by the same set of primitives, i.e. person features (Bjorkman et al. 2019; Harbour 2016; Terenghi 2023b).7In what follows, we only consider (changes in) the indexical properties of demonstrative forms; we do not instead discuss (changes in) their distribution, which is determined by discourse-pragmatic factors. Importantly, the latter is also subject to change, as part of the well-known grammaticalization cline from demonstrative forms to definiteness markers (see for instance the comprehensive discussion of this fact by Kupisch and Polinsky 2022; and, most recently for heritage languages, van Baal 2023). An anonymous TLR-reviewer asks whether we have observed any similar “overuse” of demonstrative forms: while this was not part of the original research, we acknowledge that this issue should be investigated in future research, especially as it might provide yet another case (besides that of clitic pronouns investigated in Section 6) in which syntactic change diverges for formal and interface properties. More specifically, this section reviews the patterns of change attested by ternary demonstrative systems, that is: demonstrative systems that convey three-way person oppositions, contrastively encoding proximity to the speaker (‘this near me’), proximity to the addressee (‘that near you’) and non-proximity to either (‘that far (from us)’).

These systems can be unstable in diachrony and in contact (heritage varieties, creoles) alike and, crucially, show the same patterns of change across the two domains (Terenghi 2023a, 2023b). Concretely, change in this domain amounts to simplification: both in diachrony (Table 4a) and in contact (Table 4b), ternary demonstrative systems tend to lose the contrastive hearer-oriented semantics (‘that near you’).

As both cases in Table 3 show, the contrastive semantics of the hearer-oriented demonstrative form (‘that near you’) is lost and that deictic domain is ultimately merged with the non-participant oriented one (‘that far’). This new, extended deictic domain, which substantially carries non-speaker-oriented semantics (‘that not near me’), is spelled out by the original non-participant oriented form, as exemplified by innovative Occitan varieties and by Batavia Creole in Table 4a and 4b respectively.8This new semantics may also be spelled out by the erstwhile hearer-oriented demonstrative form; for morphological variation in this respect, see Terenghi 2023b,a. Likewise, the hearer-oriented semantics may collapse with the speaker-oriented one, yielding yet another pattern of reduction in demonstrative systems; aspects of semantic variation are also discussed in Terenghi 2023a, 2023b.

**Table 3:** Demonstrative systems in diachrony (3a) and in contact (3b).

(a) Occitan demonstratives (Ledgeway and Smith 2016)			
	Near 1	Near 2	Far
Occitan (old)	aqueste	aiceste	aquel
Occitan (new)	aqueste	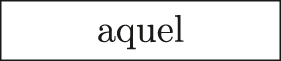	

**(b) Portuguese-based Batavia Creole (Maurer 2013)**			

	**Near 1**	**Near 2**	**Far**

Portuguese	este	esse	aquele
Batavia Creole	iste	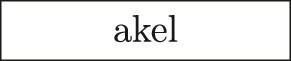	

**Table 4:** Friulian tonic and clitic subject pronouns, in Frasson et al. 2021: Table 1.

	Singular		Plural	
	Tonic	Clitic	Tonic	Clitic
1	jo	i / o	on / noaltris	i / o
2	tu	tu	vô / vualtris	i / o
3	M: lui	M: al	lôr	a / e
	F: je	F: e / a		

Comparable simplification patterns can also be identified when investigating heritage languages. Evidence for this comes from a study on demonstrative systems in heritage Sicilian and Abruzzese (Italo-Romance) varieties (Terenghi 2022). The homeland counterparts (Sicilian and Abruzzese in Italy) of the heritage varieties included in that study display a three-way deictic opposition in the demonstrative system. The heritage varieties at issue were investigated in Argentina, in Quebec, and in the United States, thus with different majority/dominant languages: respectively, Spanish, with a three-way demonstrative system comparable to the one of the homeland varieties; French, with a (reduced) two-way demonstrative system, comparable to the simplified systems discussed in Table 3; and English, with a two-demonstrative system as well. By testing heritage speakers on both production and comprehension, Terenghi concluded that the demonstrative systems of the heritage varieties under investigation are undergoing a reorganization and, more specifically, are moving towards the loss of the contrastive hearer-related semantics and therefore to a two-way opposition. However, and interestingly, this change cannot be traced back to transfer from the dominant languages, as the patterns attested in the heritage varieties do not consistently align with those shown by the respective dominant languages.

Taken together, these case studies show that the indexical oppositions encoded in demonstrative systems tend to be unstable, both in diachrony and in contact, and more specifically to undergo simplification whereby original three-way distinctions are reduced to two-way distinctions. Importantly, the same patterns of simplification are attested across CID and CIC, corroborating our conclusion that diachrony and contact behave in a similar way in the domain of indexicals.

### 4.3 Interim summary

In sum, we can conclude that CID and CIC display parallel outcomes in both pronominal and demonstrative systems. While in the former case no change is attested, in the latter comparable patterns of change are attested across the different domains (CID and CIC). This conclusion is in line with the idea that CID and CIC are two faces of the same coin, with CIC amounting perhaps to accelerated CID, as argued for by Kupisch and Polinsky (2022). However, we maintain in this paper that this conclusion is skewed by the specific phenomenon we are examining: as discussed in opening this section, personal pronouns and demonstratives can be regarded as bundles of *ϕ* features, thus as essentially syntactic items. As such, if they undergo change, they do so in line with the cognitive bias towards monotonic computations discussed in Section 3, which generally affects and constrains the outcomes of language acquisition. In the specific case of pronominal and demonstrative systems, the only possible outcome is the simplification of the system (attested for the latter) and, more concretely, the loss of the hearer-oriented semantics, as argued by Terenghi (2023b); this holds whether the languages examined are considered in isolation (but see again the discussion in Section 3.2) or in contact.

As we will show in the remainder of this paper, if we extend our discussion to phenomena which involve more knowledge of discourse (interface phenomena), the matter is considerably less straightforward and significant mismatches can be identified between the outcomes of CID and CIC.

## 5 DOM: some CIC differs from CID

Let us start by considering the case of Differential Object Marking (‘DOM’), that is: the overt morphological marking of a specific class of direct objects, usually animate, definite, specific, or highly salient, as illustrated in 1 (Diez 1882; Bossong 1985, 1991; Comrie 1989).

(1)

**Table:** 

Spanish (Fábregas 2013: 1)

a.

**Table:** 

Encontré	un	problema.
found.1sg	a	problem
‘I found a problem.’

b.

**Table:** 

Encontré	a	un	superviviente.
found.1sg	dom	a	survivor
‘I found a survivor.’

In (1a), the object is inanimate and therefore receives no marking, since animacy is the feature that triggers DOM in (Peninsular) Spanish. In contrast, (1b) shows the presence of DOM, as the object is animate. As mentioned, animacy is not the only factor that conditions DOM; we will return to the relevant distinctions below.

Although a detailed discussion of the different analyses for DOM proposed over the years would take us too far afield (but see Fábregas 2013 for a partial overview), it is safe to regard DOM as an interface phenomenon. The factors that trigger object marking cross-linguistically are in fact not (exclusively) syntactic in nature, and instead range across semantic and (discourse) pragmatic properties of the direct object (definiteness, specificity, referentiality, and animacy; and topicality) and lexical-semantics properties of the verb (Aissen 2003; Silverstein 1976; Leonetti 2008; Dalrymple and Nikolaeva 2011; López 2016; von Heusinger 2008; Klein and de Swart 2011; Khouja 2019; among many others). Typically, differentially marked objects are animate, definite, and/or specific.

Diachronic studies have shown that DOM originates in topic contexts (Iemmolo 2009, 2010, 2020); this is illustrated for instance by the following minimal pair from Old Spanish:

(2)

**Table:** 

12th century Spanish: *Cantar del mio Cid* (adapted from Iemmolo 2020: 26)

a.

**Table:** 

En	braços	tenedes	**mis**	**fijas**	tan	blancas	commo	el	sol
in	arms	hold.2pl.prs	my	daughters	so	white	as	the	sun
‘In your arms you hold my daughters as white as sun’

b.

**Table:** 

**A**	**la**	**sus**	**fijas**	en	braços	las	prendia
dom	the	his	daughters	in	arms	them=	take.3sg.pfv
‘His daughters, he took them in his arms’

As (2) shows, one and the same human, animate, and definite direct object (*fijas* ‘daughters’) is not preceded by the DOM-marker *a* ‘to’ when *in situ*, as in (2a), but it is preceded by the DOM marker *a* when fronted, as in (2b). Importantly, in present-day Spanish a similar human, animate, and definite DP is also differentially marked *in situ*: that is, the differential marker *a* undergoes an extension in diachrony, from being restricted to topic contexts only to being used for *in situ* marking as well. According to Dalrymple and Nikolaeva (2011: 2), this is a case of grammaticalization: the semantic features that trigger the differential marking of the object in present-day Spanish, as well more widely cross-linguistically (namely animacy, definiteness, and specificity) are in fact prototypically associated with topics.9One anonymous TLR-reviewer asks about the extent to which DOM can be considered an interface phenomenon nowadays. In Spanish DOM is certainly not only a marker of topicality, since it is now commonly used for any animate (and definite, in the case of Rioplatense Spanish, for instance) object. In Spanish, topicality and animacy have converged into the same morpheme, but this does not exclude necessarily that DOM is not also acting as a topic marker, in addition to being an animacy marker. According to Dalrymple and Nikolaeva (2011: 2): “marked objects are associated with the information-structure role of topic. The association may be either synchronic or historical. Where the direct connection between marked objects and topicality has been lost through grammaticalization, marked objects in some languages become associated with semantic features typical of topics (animacy, definiteness, specificity).” We interpret this as indicating that the two functions are not mutually exclusive. This path of change has been shown to generally hold cross-linguistically (Iemmolo 2010, 2020; among others).

In contact, however, DOM has been reported to weaken or even to disappear; the most extensively investigated case of DOM-loss in contact settings is provided by heritage varieties, and in this respect heritage Spanish spoken in the US has been studied extensively (Luján and Parodi 2001; Montrul 2004; Montrul and Bowles 2009; Montrul et al. 2015; Montrul and Sánchez-Walker 2013; Silva-Corvalán 1994). These findings do not hold exclusively for Spanish; for instance, the same outcome is attested in southern Italo-Romance varieties spoken in New York City, as shown by the data from heritage Sicilian and heritage Cilentano in (3), from Andriani et al. (2022b: 17):

(3)

a.

**Table:** 

Vo’	canosciàre **Ø**	**u**	**pecceriɖɖu**
want.1sg	know.inf	the	child
‘I want to know the child’ (Heritage Sicilian in New York City)

b.

**Table:** 

Io	conosciuto **Ø**	**tuttəquandə**
I	met	everyone
‘I’ve met everyone’ (Heritage Cilentano in New York City)

Although the homeland varieties of Sicilian and Cilentano display DOM with human referents (*pecceriɖɖu* ‘the child’ and *tuttəquandə* ‘everyone’ in (3); Ledgeway 2018), the examples in (3) confirm that the marker is dropped in the heritage context, as indicated by ‘Ø’.

This abbreviated review of the available research concerning how DOM changes in diachrony and in contact seem to falsify Kupisch and Polinsky’s (2022) conclusions: in fact, the two paths of change look diametrically opposed, with the emergence of DOM in diachrony (spreading from topicalized objects to regular *in situ* objects), and the loss of DOM in *in situ* contexts in heritage varieties (that is, in contact). In this sense, the behavior of DOM in contact is a clear challenge for the equation of change in contact to change in diachrony.

However, this conclusion is too hasty: once we consider different contact situations, in fact, we can observe different CIC-outcomes for DOM. In what follows, we consider microcontact data (see again Section 3.2), which suggest that the development of DOM in contact may also closely resemble the diachronic one. Indeed, DOM can be shown to emerge in these specific contact situations, contrary to what can be concluded from (3). Importantly, in these contact varieties DOM emerges in topic contexts and then gradually extends to *in situ* objects, on a par with the conclusions of the diachronic literature on the topic.

Similar conclusions can be drawn from some moribund heritage varieties of German spoken in the US and in Argentina which, despite not being in microcontact, show the same behavior that we observed for the Romance languages in contact (Yager et al. 2015). Investigating samples of speech from diasporic varieties of German spoken in Misiones, Argentina, Texas, and Wisconsin, Yager et al. (2015) show that in lieu of the collapse of the dative case in these varieties, remaining speakers produce dative forms that align with established DOM-patterns. Agreeing with these authors, we interpret this shift as a reanalysis of the realization of case features in these grammars to express topicality (i.e., discourse functions).

Finally, and again from the heritage domain, some Italo-Romance and Rhaeto-Romance heritage varieties spoken in Argentina and Brazil likewise show the emergence of DOM. Here we illustrate this by means of data from heritage Friulian. Homeland Friulian is a Rhaeto-Romance variety that does not display DOM *in situ* and may only mark dislocated object pronouns. However, fieldwork data collected with heritage Friulian populations in Argentina consistently displayed DOM-effects in topic context with full DPs, as in (4a), and, although rarely, some possible cases of DOM *in situ* were also elicited, as in (4b):

(4)

**Table:** 

Heritage Friulian in Argentina (from Andriani et al. 2022b: 21, 19)

a.

**Table:** 

**A**	**une**	**cjantant**,	îr,	la=ai	bussade
DOM	a	singer	yesterday	her=aux	kissed
‘As for a singer, I kissed one yesterday’

b.

**Table:** 

An	clamat	**a**	**me**	**mari**
have.prs.3pl	called	DOM	my	mother
‘They’ve called my mother’

As shown in the foregoing discussion, DOM in microcontact scenarios show the same syntactic development as in diachrony, whereas DOM in other types of contact results in different outputs: while the latter cases involve simplification (loss of DOM, loss of indeterminacy), microcontact , just like diachrony, involve complexification (slow emergence of DOM from topic contexts, creation of indeterminacy). Said otherwise, we cannot straightforwardly maintain that change in contact is simply accelerated diachronic change, as regards DOM: while this is true in some contact contexts (here: microcontact), the outcomes of change in contact and in diachrony are not convergent in other contact contexts. In the next Section, we review another case in which the relation between change in contact and change in diachrony is likewise not fully isomorphic.

## 6 Subject clitics: disentangling *ϕ*-features and discourse

This final section takes a closer look at subject clitics (‘SCls’) and compares their development in diachrony and in contact. The presence of subject clitics, i.e. reduced, unstressed pronominal forms, characterizes many northern Italian varieties. As an example, Table 4 displays the Friulian pronominal system containing both tonic pronouns and subject clitics.

Northern Italian SCLs are particularly telling with respect to the difference between CIC and CID. Here, we will be considering the SCls systems of Northern Italo-Romance varieties, and in particular Venetan ones.10In what follows, we exclusively focus on proclitic SCls and instead leave enclitic SCls for future research. The latter set of clitics may be morphologically distinct from the former and is restricted to some syntactic environments (e.g. interrogatives; see Poletto and Tortora 2016 for an overview). SCls differ from full pronouns in a number of ways. First, SCl paradigms may present gaps and these gaps can be modelled along an implicational hierarchy, as originally observed by Renzi and Vanelli (1983: 143–144):11A handful of exceptions have been identified by Poletto (2000) and by Manzini and Savoia (2005), but (5) is a solid trend.

(5)

**Table:** 

2sg < 3sg < 3pl < 1sg, 1pl, 2pl

As indicated by (5), if a northern Italo-Romance variety has only one SCl, it will have a 2nd person singular one; if it has only two SCls in its paradigm, they will be the forms for 2nd and 3rd person singular; and so on.

Second, unlike full pronouns, and despite not being present in all possible configurations in every variety (see again (5)), SCls are obligatory in certain syntactic contexts. Concretely, Poletto (2000) shows that the distribution of clitics varies along a scale, ranging from varieties where SCls double variables, QPs, full DPs, and pronouns, to varieties where they double only full pronouns. The syntax of Venetan SCls is particularly interesting in this respect, as their distribution is determined both by the (generally acknowledged) purely syntactic factors and by recently identified discourse factors (Frasson et al. 2021; Frasson 2022).12Specifically, Frasson argues that the distribution of pronominal elements depends on how strongly referential a given pronoun is, that is, on whether that pronoun can be used to switch the reference to a non-salient discourse antecedent. This switch can only be performed if the pronoun encodes the referential feature [uR], which is typically present in strong pronouns (used in topic-switch contexts) and absent in weak ones (not used in topic-switch contexts). Frasson’s (2022) proposal is that also clitic pronouns *may* carry [uR]; when this is the case, their distribution is similar to that of strong pronouns. Despite these points of microvariation, nonetheless, we can assert that SCls cannot be freely dropped.

Third, SCl paradigms often exhibit syncretic forms, which further sets them apart from full pronoun paradigms. Because of these characteristics, SCls are often considered more akin to verbal inflection than to pronouns (see Poletto and Tortora 2016 for a thorough description of these elements).

Recall now that our hypothesis on syntactic change is that CID mirrors CIC only as far as *ϕ*-features are concerned, but that their outcomes may diverge when multiple modules of grammar must be integrated. As such, SCls, and especially SCls in Venetan varieties, are the perfect testing ground for our hypothesis, in that they exhibit some clear generalizations linked to the paradigm, and therefore to their *ϕ* features, and some others linked to their (discourse-oriented) distribution. Based on our heuristic, we thus predict that the output of CID and CIC targeting the *forms* of the paradigm (*ϕ* features) will be the same; instead, changes affecting the *distribution* of SCls will possibly differ across CID and CIC, since distribution facts depend, among others, on discourse factors.

In what follows, we demonstrate that our predictions are borne out. First, we discuss the parallelism between CID and CIC when paradigm *forms* undergo change (Section 6.1). Next, we turn to the divergence between the outcomes of CID and CIC, which is attested when change targets the *distribution* of SCls (Section 6.2). Further, we show that, in this latter case, different contact situations may have different outcomes: this strongly suggests that the outcome of CIC becomes less predictable on the basis of CID, where discourse elements are concerned.

### 6.1 Subject clitics and *ϕ*-features: CIC equals CID

The diachronic development of SCls represents an instance of the subject agreement cycle, in which full pronominal expressions are reanalyzed as agreement markers and eventually fall out of use, first identified by Givón (1976). More recently, van Gelderen (2009, 2011) has provided a minimalist account for the subject agreement cycle in terms of Feature Economy:

(6)

**Table:** 

**Feature Economy**
Minimize the semantic and interpretable features in the derivation
(van Gelderen 2011: 20)

Concretely, the interpretable *ϕ*-features of full pronouns are reanalyzed as uninterpretable *ϕ*-features during acquisition, leading to their realization as agreement markers; these may subsequently undergo loss altogether:

(7)

**Table:** 

Adjunct	>	Specifier	>	Head	>	ø
emphatic		full pronoun		agreement		—
[semantic]		[iF]		[uF]		—
(modified from van Gelderen 2011: 41)						

This formalization is consistent with the diachronic literature on SCls in northern Italo-Romance varieties. Concretely, and as already argued in the foregoing section, SCls in northern Italo-Romance varieties can be regarded as syntactically akin to agreement markers, unlike full pronouns.13Note that two different types of SCls can be distinguished on syntactic *vs* phonological grounds: the northern Italo-Romance-type and the French-type respectively (Brandi and Cordin 1989; Rizzi 1986). The former represent a more advanced stage along the cycle (but see Renzi’s (1992) analysis for innovative French varieties); however, we will not be addressing this point further here in the interest of space and our discussion will simply refer to the northern Italo-Romance type. Further, the diachronic emergence of SCls following the reanalysis of erstwhile independent pronouns has been carefully traced in a large body of research for a diverse set of Italo-Romance varieties (Poletto 1995; Renzi 1992; Parry 1993; Vanelli 1987; Spiess 1956; Vanelli 1984; among many others). Given our focus, it is worth pointing out here that Poletto (1995) shows that northeastern Italo-Romance SCls originated from a set of independent pronouns between the 16th/17th century.

Against this rich background, substantially less research has focused on the final part of the cycle, namely the diachronic loss of SCls. Before proceeding, a disclaimer is in order: cliticization of erstwhile free subject pronouns targeted different pronouns to different extents, and possibly not reaching a full paradigm of SCls. This state of affairs was discussed, for example, for the Venetan dialect of Verona by Pescarini (2022). As such, present-day incomplete SCl paradigms need *not* be the diachronic result of the erosion of original larger paradigms; they might instead have never been complete SCl paradigms. This notwithstanding, there is evidence that SCl paradigms underwent a reduction process in diachrony across different northern Italo-Romance varieties. Importantly, the erosion of SCl paradigms is consistent with the implicational hierarchy in (5): that is, the SCls for 1sg, 1pl, and 2pl are more prone to falling out of use over time than the SCls for 3pl, 3sg, and even more so: than 2sg. Direct evidence for this comes from, among others, Milanese (Vai 2018: 167), Genoese (Scala 2021: 10), and central Venetan varieties (Poletto 1993: 156, 171). Despite different timelines (some paradigms underwent reduction in the 18th century, others in the 19th century), we can identify the new, reduced paradigms as the outcomes of CID.

A similar erosion of SCl paradigms is also attested more recently in other varieties, for example across Piedmontese varieties (Scala 2021: 12) or in Florentine (Renzi 1992: 95). Furthermore, clitics for 1sg, 1pl, and 2pl are commonly reported to be optional across various northern Italo-Romance varieties, again in line with the diachronic facts discussed above. Crucially for our discussion, the attested optionality and the eventual loss of SCls are commonly linked to the influence of Standard Italian, which itself lacks SCls (see e.g. remarks in Goria 2004: 159 for Turinese and Astigiano).

In sum, when it comes to the *forms* of the paradigm, CIC and CID affect the clitic paradigm in the same way, as in both cases the reduction of the paradigm is compatible with the implicational hierarchy uncovered by Renzi and Vanelli (1983). Tying this back to our heuristic for change, this means that the *ϕ*-features that underlie the derivation of the different SCl forms are affected by change, as some person and/or number distinctions in the paradigm are lost;14Note that whether all SCls or only a subset thereof carry *ϕ* features is insubstantial to our argument. So far we have implicitly regarded SCls as encoding *ϕ*-features across the board, contrary to the formalization proposed by Poletto (2000: chapter 2) under which only 2nd and 3rd person SCls carry (a combination of) person and/or number features. However, and crucially, the cases of paradigm erosion discussed in the foregoing all include at least one of the person- or number-carrying SCls in Poletto’s (2000) analysis, thus fully justifying our reference to *ϕ* features in this domain even under more restrictive assumptions about the distribution of *ϕ* features within the SCl paradigm. but that they are affected by change in one and the same way, as the patterns of reduction of the paradigms are parallel across CID and CIC. This parallelism is predicted by our proposal, since we are dealing exclusively with syntactic features. However, when we turn to observe the *distribution* of SCls, which is in part determined by discourse factors, the entire picture changes drastically: we explore this in the next section.

### 6.2 Subject clitics and discourse: CIC differs from CID

Granted that SCl paradigms come in different sizes across Italo-Romance varieties (possibly due to the diachronic or contact-induced erosion of the relevant paradigms discussed in the previous section), in SCl-languages SCls are expected to occur in some specific syntactic environments and under specific discourse conditions (see the introduction to Section 6). In what follows, we illustrate three changes that affect the *distribution* of SCls in northern Italo-Romance. All these developments have emerged *in contact* (with Standard Italian, Brazilian Portuguese, and Argentinian Spanish respectively) and crucially find no correspondence in diachrony.

The first effect of contact on the distribution of SCls is a general restructuring of the conditions of occurrence of SCls, leading to these forms being absent in various syntactic and pragmatic environments in which their occurrence is expected, and vice versa present in environments in which their occurrence is not expected. Importantly, this change cannot be simplistically traced back to transfer from SCl-less Standard Italian, but it should be regarded as an instant of CIC nonetheless, as it stems from the multilingual competence of the relevant speakers. For example, Casalicchio and Frasson (2018) investigated change-in-progress in the distribution of SCls in present-day central Venetan varieties. In central Venetan varieties, SCls double both full pronouns and topicalized referential DPs in preverbal position (Benincà 1982, 1983; Poletto 2000).

The results of Casalicchio and Frasson’s acceptability study indicate that, while older speakers substantially adhere to this grammar, younger speakers tend to use SCls according to a syntacticized version thereof. Specifically, the innovative grammar seems to be driven by a general simplification of the interface requirements on the use of SCls: SCls appear to be prone to omission in contexts in which their occurrence is regulated by syntax-pragmatic interface factors, and concretely when they double a topicalized DP subject; on the contrary, SCls appear to be more freely accepted by younger speakers in contexts in which a preverbal subject is missing (atmospheric verbs and postverbal subjects with unaccusative verbs). That is, the innovative grammar seems to rely exclusively on syntactic rules (insert a SCl if the preverbal subject position is not already filled), at the expenses of the formerly available interface conditions (topicality for the syntax-pragmatic interface; argument structure in the case of atmospheric and unaccusative verbs for the syntax-semantics interface).

The second outcome of CIC that we can observe regards the distribution of SCls and null subjects. As discussed by Frasson (2021) for heritage Venetan spoken in Brazil and by Frasson et al. (2021) for heritage Friulian spoken in Argentina and Brazil, heritage subject clitics do not disappear, unlike their homeland counterparts (see above), but rather come to display a full pronominal behavior. This conclusion is based on the fact that these erstwhile agreement markers do not pass Rizzi’s (1986) tests for clitichood any more: for instance, they can be dropped under coordination in contexts (9) in which this would not be possible in the baseline (8):

(8)

**Table:** 

Trevigiano (Frasson 2021: 7)				
Te	canti	e	*(te)	bali.
you.scl	sing.2.scg	and	you.scl	dance.2.sg
‘You are singing and dancing.’

(9)

**Table:** 

Brazilian Venetan (Flores Da Cunha; Frasson 2021: 10)					
I	riva	e	sfrunha	el	teren.
they.scl	come.3pl	and	rummage.3pl	the	ground
‘They come and rummage the ground.’					

As discussed in the foregoing, in the baseline SCls are agreement markers and as such an integral part of the verbal morphological system. Therefore, whenever two clauses are coordinated, SCls must be present in both conjuncts, as shown in (8). This is not the case for full pronouns, which are instead fully referential elements and not inflection markers. Full pronouns can thus be dropped under coordination even in languages that are not *pro*-drop, provided that the subject of the two clauses is the same. The fact that in the Brazilian Venetan example in (9) the subject is only overtly realized in the first conjunct and is missing in the second one plainly suggests that these elements degrammaticalized in heritage varieties and reverted to full pronominal status. This conclusion could not be foreseen on the sole bases of diachronic change, where the subject agreement cycle proceeds instead to completion.

Interestingly, Frasson et al. (2021) show that these pronominalized SCls tend to be dropped in topic continuation contexts, as partly shown by (9) and better exemplified in (10):

(10)

**Table:** 

Friulian (Buenos Aires; Frasson et al. 2021: 26)														
Gno	santul	al	me	a	dite: […]									
my	godfather	he.scl	me.ocl	has	said									

a.	shift													
	I	ai	tacat	fevelà	in	furlan, […]								
	I.scl	have	started to	speak	in	Friulian								

b.	continuation													

	[…]	dop	*pro*	ai	vut	ancje	la	fortune	di	sposà	une			
		then	pro	have	had	too	the	fortune	to	marry	a			
	fie	di	furlans.											
	daughter	of	Friulians											
‘My godfather told me: […]. I started to speak Friulian, […] then I was lucky enough to marry a Friulian descendent.’														

(10) displays a clause introducing a new referent (the godfather): this is the new topic in the wider discourse, and as such this element is also marked with a 3sg SCl. The sentence continues and we can see that in (10a) the topic shifts with respect to the previous clause: the new topic is the speaker, which is marked by the SCl for 1sg. However, in the absence of further topic shifts (that is, under topic continuation, as in (10b)), we see that the SCl is dropped. Frasson et al. (2021: 26) found this pattern, whereby SCls are produced in topic shift and dropped in topic continuation, to be significant in Argentina; a similar effect of topicality, albeit a non-statistically significant one, was found in Brazil.

These facts are surprising for two reasons: the first one is that, when Brazilian Portuguese is the contact language, this effect cannot be attributed to transfer from the contact language. Brazilian Portuguese is in fact a partial null-subject language. More interestingly, heritage speakers are known to have difficulties mapping meaning onto silence (the Silent Problem, first identified by Laleko and Polinsky 2017): accordingly, heritage speakers of null-subject languages have been found to disprefer null subjects (see Polinsky 2018: 6.5 for an overview). Therefore, the fact that a heritage language starts developing a null subject is all the more surprising (but see Carvalho and Child 2011, Pérez 2009, Souza et al. 2018 for some attested cases of emergent pro-drop). Focusing now on SCls, this evolution is also interesting as it diverges slightly from the baseline Veneto. As shown by Benincà (1994), Veneto is a language where SCL are only licensed with overt subjects if the subjects are topics, i.e. almost exactly the same contexts in which their heritage counterpart drops them.

A third and final context in which the development of SCls in diachrony diverges from that in contact is the case of 3rd person SCl. Heritage Venetan and Trentino varieties spoken in Brazil display an interesting case of complexification in relation to these new auxiliary forms, discussed in D’Alessandro and Frasson (2023). Not only do SCls not get lost, as we would expect in diachrony, but original patterns of diatopic variation are reinterpreted yielding novel specialized auxiliary + SCl elements. Auxiliaries in Veneto come in three fashions: *l’è*, *z’è*, *è*. These three variants are geographically distributed (Benincà 2007; Bentley et al. 2015; Poletto 1993), and consist in two cases of a SCl (*l, z*) incorporated into the 3rd person singular form of the auxiliary *be*. In Brazil, however, these “complex” auxiliaries specialized, as shown in (11), from D’Alessandro and Frasson (2023):

(11)

a.

**Table:** 

L’=è	(*zè	/ *è)	vegnesto	la	nona
scl=be.3sg.prs	scl.be.3sg.prs	/ be.3sg.prs	come.ptcp	the	grandmother
“My grandmother came here”

b.

**Table:** 

La	so	mare	zè	(*l’è	/ *è)	nasesta	in	Italia
the	his	mother	scl.be.3sg.prs	scl.be.3sg.prs	/ be.3sg.prs	born	in	Italy
“His mother was born in Italy”

c.

**Table:** 

I	noni	è	(*l’è	/ *zè)	vegnesti
the	grandparents	be.3sg.prs	scl=be.3sg.prs	/ scl.be.3sg.prs	come.ptcp
de	navio				
of	boat				
“The grandparents have come by boat”

(11) illustrates the specialization patterns: *l’è*, in (11a), is used by preference with postverbal subjects and anti-agreement effects; *zè*, in (11b), is used by preference with preverbal subjects and no anti-agreement effects; finally, *è*, in (11c), is used by preference with preverbal third person plural subjects.15Other combinations are only marginally attested.

What happened in Heritage Venetan in Brazil is that these auxiliaries with incorporated clitics came to be part of the same “pool of variants” (Labov 1972) for a group of speakers originating from various parts of Veneto. These speakers did not select one form and abandon all others to simplify the system, but created a distribution rule, as illustrated, assigning one auxiliary + SCl form per construction. For instance, *l’è* is used almost exclusively with postverbal subjects and is associated to anti-agreement effects. While these forms are not strictly speaking purely SCl, they involve SCl in a such a way that integrates them in a highly complex syntactic system not found in the varieties spoken in Italy.

In conclusion, we hope to have shown that when only *ϕ*-features are involved, SCl in contact do not preset major differences from SCl that evolve spontaneously. However, when discourse or more complex syntactic computation is involved, the output of CIC suddenly differs substantially from CID, proving that a generalization along the lines of “CIC is accelerated CID” only holds for “core” parts of grammar, not those concerning the interfaces.

## 7 Conclusions

Our review of phenomena taken from various domains of syntax, from purely structural ones to those that sit at the interface, shows that we cannot make any wide-ranging generalizations as to the relations between syntactic change in diachrony and in contact. To summarize our findings:

– Pronouns and demonstratives appear to behave similarity in contact- and diachronic-settings;
– DOM development is rather unpredictable, due to it being largely dependent on discourse;
– Subject clitics present a split behavior: the part that is strictly related to *ϕ* features shows a perfectly parallel behavior between CIC and CID; the clitic domain more related to their *distribution*, which can be shown to be linked to discourse, presents very clear divergence between CIC and CID.

Taken together, these findings suggest that *ϕ*-features and discourse-features develop differently over the course of time (in the case of diachronic developments) versus contact-environments. Demonstratives and *ϕ*-features show some degree of sensitivity to monotonicity (Terenghi 2022, 2023a, 2023b), resulting in more predictable trajectories and paths of development. In contrast, ‘interface’ phenomena that are more reliant on topicality and discourse-features are more difficult to predict, and as we have argued here, not always on par with previously observed and documented diachronic developments.

In closing, we offer a tentative explanation for this distinction. Sequences of features with binary values adhere to monotonic sequencing. The moment this harmonic ordering of a monotonic sequence is disrupted, a change will occur. Monotonic sequences by their very nature are stable, and the conflicting types are those where change starts in both diachrony and in contact settings; e.g., the realization of second person and duals. Features and the monotonic ordering offer a fundamental explanation here. The situation radically differs when we home in on interface phenomena, such as DOM. What is relevant for DOM is topicality and *not* the realization of *ϕ*-features (not primarily, at least). In similar fashion, the emergence of null subjects in microcontact situations is closely related to topicality conditions; i.e., while languages in contact situations have been found to ‘lose’ – or perhaps my aptly stated, ‘significantly reduce’ – pro-drop and overt subjects, subject clitics are eliminated in microcontact. A factor that cannot be overlooked is that these subject clitics at some point in their development became fully pronominal, meaning that they are could stand on their own. It is the fully pronominal forms of these elements there were eventually dropped in the appropriate discourse/topic context. The same situation holds for DOM in Romance varieties in Argentina, due to its function as a linker to discourse context. The reported rise in DOM-like structures in lieu of dative case marking in some moribund varieties of heritage German in Argentina, Texas, and Wisconsin opens the door to questions concerning their connection with oblique case features with discourse functions in contact settings (Yager et al. 2015). We leave this fascinating question for future research.

In conclusion, we have shown that CIC and CID have comparable outputs and can therefore be considered based on one underlying mechanism only as far as the *ϕ*-characteristics are concerned. However, if discourse features are involved, the outcome of CIC differs radically from that of CID. Moreover, while CID is largely predictable, CIC is not: the evolution of syntactic rules involving discourse features can be varied, and it ranges from simplification to complexification.
